# Novel KRAS Gene Mutations in Sporadic Colorectal Cancer

**DOI:** 10.1371/journal.pone.0113350

**Published:** 2014-11-20

**Authors:** Walid M. Naser, Mohamed A. Shawarby, Dalal M. Al-Tamimi, Arun Seth, Abdulaziz Al-Quorain, Areej M. Al Nemer, Omar M. E. Albagha

**Affiliations:** 1 Molecular Diagnostics Lab, Department of Laboratory Medicine, King Fahd Hospital of the University, University of Dammam, Al-Khobar, Saudi Arabia; 2 Pathology Department, College of Medicine, University of Dammam, Dammam, Saudi Arabia; 3 Molecular Diagnostics, Department of Anatomic Pathology, Sunnybrook Health Sciences Centre, Toronto, Ontario, Canada; 4 Department of Internal Medicine, College of Medicine, University of Dammam, Dammam, Saudi Arabia; 5 Rheumatology Section, Centre for Genomic and Experimental Medicine, MRC Institute of Genetics and Molecular Medicine, University of Edinburgh, Western General Hospital, Edinburgh, United Kingdom; Odense University hospital, Denmark

## Abstract

**Introduction:**

In this article, we report 7 novel KRAS gene mutations discovered while retrospectively studying the prevalence and pattern of KRAS mutations in cancerous tissue obtained from 56 Saudi sporadic colorectal cancer patients from the Eastern Province.

**Methods:**

Genomic DNA was extracted from formalin-fixed, paraffin-embedded cancerous and noncancerous colorectal tissues. Successful and specific PCR products were then bi-directionally sequenced to detect exon 4 mutations while Mutector II Detection Kits were used for identifying mutations in codons 12, 13 and 61. The functional impact of the novel mutations was assessed using bioinformatics tools and molecular modeling.

**Results:**

KRAS gene mutations were detected in the cancer tissue of 24 cases (42.85%). Of these, 11 had exon 4 mutations (19.64%). They harbored 8 different mutations all of which except two altered the KRAS protein amino acid sequence and all except one were novel as revealed by COSMIC database. The detected novel mutations were found to be somatic. One mutation is predicted to be benign. The remaining mutations are predicted to cause substantial changes in the protein structure. Of these, the Q150X nonsense mutation is the second truncating mutation to be reported in colorectal cancer in the literature.

**Conclusions:**

Our discovery of novel exon 4 KRAS mutations that are, so far, unique to Saudi colorectal cancer patients may be attributed to environmental factors and/or racial/ethnic variations due to genetic differences. Alternatively, it may be related to paucity of clinical studies on mutations other than those in codons 12, 13, 61 and 146. Further KRAS testing on a large number of patients of various ethnicities, particularly beyond the most common hotspot alleles in exons 2 and 3 is needed to assess the prevalence and explore the exact prognostic and predictive significance of the discovered novel mutations as well as their possible role in colorectal carcinogenesis.

## Introduction

The development of cancer (carcinogenesis) is a multistep process that is believed to result from accumulation of genetic alterations in a single cell during the life of an individual. The number of genes found to be associated with different cancers is growing rapidly. The most frequently activated genes are members of the RAS gene family, two of which (Harvey- H) and (Kirsten-K) were first identified as being homologous to viral transforming genes, while the third (NRAS) was identified in a human neuroblastoma. The RAS gene product is a monomeric membrane-localized G protein of 21 kd that functions as a molecular switch linking receptor and non-receptor tyrosine kinase activation to downstream cytoplasmic or nuclear events. Each mammalian cell contains at least three distinct RAS proto-oncogenes encoding closely related, but distinct proteins. RAS genes can be activated by various point mutations including those affecting codons 12, 13, 61, or 113–117. Signal transduction by ras proteins involves hydrolysis of GTP, and activating mutations inhibit this process, locking the protein in the “on” signaling conformation [1]. Activating mutations in these RAS proteins result in constitutive signaling, thereby stimulating cell proliferation, and inhibiting apoptosis.

Oncogenic KRAS mutations are prevalent in virtually all cancer types, making KRAS one of the most frequently mutated genes in human cancers [2]. The RAS oncogenes, together with the p53 tumor-suppressor gene, are the genes most consistently found to be mutated in colorectal cancer [3]–[4] in which epithelial cells of the colorectum progress from small adenoma through large adenoma, and finally become adenocarcinoma [3].

Numerous studies have reported the frequency of KRAS gene mutations in colorectal cancer. The largest series was the “RASCAL” study of 2721 colorectal cancer cases reporting an incidence of 37.7% [5]. Comparable frequencies were reported subsequently [6]–[8].

The aim of this article is to report seven novel KRAS mutations and open the door for further KRAS testing on a large number of patients in order to assess the prevalence and explore the exact prognostic and predictive significance of the discovered mutations as well as their possible role in colorectal carcinogenesis. The novel mutations were discovered while retrospectively studying the prevalence and pattern of KRAS mutations in cancerous tissue obtained from 56 Saudi sporadic colorectal cancer patients from the Eastern Province, and correlating these with clinical features, and p53, EGFR (epidermal growth factor) and HER2 (human epidermal growth factor receptor2) protein expression, and EGFR gene mutational status. It was not our intention to explore all possible KRAS mutations that may be found in colorectal cancer, so we just targeted the mutations that are commonly involved in that disease. The discovery of new mutations came just by chance while performing the study. To the best of our knowledge, this is the first study on the pattern of KRAS gene mutations in Saudi colorectal cancer patients.

## Materials and Methods

### Ethics statement

The study was approved by the Standing Committee of Research Ethics on Living Creatures (SCRELC) which is the IRB of our institution (Approval Number: IRB-2014-01-324). It was not possible to obtain written informed consent from participants as most of the patients were lost to follow up. This issue was discussed with the committee which concurred that, in keeping with the “Guidelines for Ethical Research Practice” [9], consent is not required in such a retrospective study as the information utilized by the authors was already accessible to the public and did not disclose patient identity. Moreover, the study carried no risk to the participants.

Fifty-six sporadic colorectal carcinoma cases of Saudi patients were retrieved from the archives of the Pathology Department of King Fahd Hospital of the University. The cases were randomly selected based on the availability of clinical data and representative paraffin tissue blocks sufficient to perform the required procedures, as well as negative family history for colorectal cancer. The time frame covered was 11 years (1998–2008).

### Clinical and pathological data

Clinical and pathological data including histologic type, grade and stage of the cancer were collected from the patients' records. Clinical and pathological data were classified using WHO criteria [10].

### Immunohistochemical staining

Immunohistochemical staining was performed for EGFR, HER2 and p53 on 4 µm-thick paraffin sections cut from blocks of colorectal carcinomatous tissue. The staining was performed in a Ventana Benchmark automated immunostainer according to the manufacturer's instructions (Ventana Medical Systems Inc., Strasbourg). Sources and dilutions of the primary antibodies used in the study are shown in Table S1. The immunostained sections were examined under a light microscope and evaluated manually by two pathologists. Definite membrane and/or cytoplasmic staining was necessary to consider a case as EGFR or HER2/neu positive, while nuclear staining of at least 10% of the cancer cells was needed for p53 positivity (Figure S1).

### Genomic DNA extraction

Five to eight 10 µm-thick formalin-fixed, paraffin-embedded cancerous tissue sections were used to extract genomic DNA. Sections were deparaffinized twice for 5 minutes in xylene, sequentially rehydrated in 100, 96 and 70% ethanol for 30 seconds each, stained with haematoxylin for 30 seconds, rinsed with water and incubated overnight in 1 M NaSCN at 37°C to remove cross-links. Slides were rinsed twice for 10 minutes in 1× PBS at room temperature, and completely air-dried. As indicated by an experienced pathologist, cancerous tissue was scraped from the glass slide surface with a scalpel to obtain at least 70% tumor cells and then transferred to 2.0 ml micro-centrifuge tubes. Genomic DNA was then extracted using the WaxFree DNA Kit (TrimGen, Maryland) implementing the standard protocol described by the manufacturer with overnight incubation in step 14 of the protocol. Extracted genomic DNA was quantified with Epok spectrophotometer and analyzed by 0.8% agarose gel electrophoresis to visualize DNA size distribution.

For those patients who exhibited novel KRAS exon 4 mutations, we also extracted genomic DNA from noncancerous colorectal tissue as described above. The noncancerous tissue was obtained from a tumor free resection margin from the colectomy specimen of the same patient.

### PCR amplification of KRAS exon 4

Sequencing was applied to detect KRAS exon 4 mutations as kits for detection of A146T - which is the exon 4 mutation reported to be commonly involved in colorectal cancer [11]–[12] - were not commercially available. A nested PCR approach similar to that adopted by Fadhil, et al [13] was implemented to amplify and perform melting analysis for exon 4 of the KRAS gene in order to check for successful PCR reactions and specificity. The first PCR round was undertaken in a final volume of 25 µl. Each reaction contained 1× QIAGEN Multiplex PCR Master Mix, 0.5× Q-solution, 2 primer pairs (Table 1) covering hotspots in KRAS exon 4 with 0.3 µM final concentration for each primer and 10 ng template DNA. PCR was performed using ABI's Veriti Thermal Cycler (Life Technologies) for 15 minutes at 95°C, 40 cycles each of 30 seconds at 94°C, 90 seconds at 55°C and 90 seconds at 72°C, followed by 10 minutes at 72°C. After addition of 2 µl (5 x) loading dye, 10 µl of each amplified sample was electrophoresed on a 2% TBE agarose ethidium bromide-stained gel and visiualized with GelDoc system from Biorad.

**Table 1:** Primers used for KRAS exon 4 mutation analysis.

Primer Name	Primer Sequence	Amplicon Size	Purpose
KRAS4-F	5′-AGACACAAAACAGGCTCAGGA-3′	160 bp	1^st^ round PCR
KRAS4-R	5′-TTGAGAGAAAAACTGATATATTAAATGAC-3′		
NKRAS4-M13F	5′-tgtaaaacgacggccagtGACACAAAACAGGCTCAGGACT-3′	105 bp	Nested-PCR
NKRAS4-M13R	5′-caggaaacagctatgaccCAGATCTGTATTTATTTCAGTGTTA-3′		
M13-F	5′- tgtaaaacgacggccagt -3′		Sequencing
M13-R	5′- caggaaacagctatgacc -3		

High resolution melting curve analysis (HRM) was performed using nested PCR reaction in a final volume of 15 µl, which contained 1× HRM Master Mix (Qiagen) and 1 primer pair specific for exon 4 (Table 1) with each primer at 0.7 µM final concentration. The template consisted of 1.5 µl of a 1∶100 dilution of the product from the first round PCR reaction. Nested PCR reaction was performed as described in the instruction manual of the HRM Type-it Kit (Qiagen).

HRM is used to assess the dissociation (melting) characteristics of double-stranded DNA (dsDNA). Base-pair composition and GC content influence melting behavior of different dsDNA fragments. During HRM, dsDNA is exposed to gradually increasing temperature in the presence of a fluorescent dye (e.g. EvaGreen). The fluorescent dye fluoresces only when bound to dsDNA. Fluorescence is monitored during transition of dsDNA to single-stranded DNA (ssDNA) which decreases as dsDNA melts. Plotting changes in fluorescence after generating specific DNA fragments during PCR give rise to single peaks corresponding to specific amplicons [14].

### DNA sequencing

After HRM, successful and specific PCR products showing one melting peak were column purified using the QIAquick PCR Purification Kit (Qiagen) according to the manufacturer's instructions. The PCR products were eluted with 30 µl elution buffer and diluted 1∶10 with water. Diluted PCR products were then used as template for cycle sequencing via Big Dye Terminator v1.1 kit (Applied Biosystems). Bidirectional sequencing (i.e. forward and reverse) was performed using cycle sequencing reaction (10 µl final volume) consisting of 1× terminator premix, 1× sequencing buffer, 0.4 µM of either M13-F or M13-R primers (Table 1) and 4 µl of diluted template. The reactions were run on Veriti (Life Technologies) according to the following protocol: One cycle of 95°C for 15 minutes; 30 cycles of 95°C for 10 seconds, 55°C for 5 seconds, 72°C for 4 minutes. Sequencing reactions were purified with ABI's BigDye XTerminator Purification Kit (Life Technologies) and loaded on a 3500 Genetic Analyzer (Life Technologies). Sequencing data were analyzed using Sequencing Analysis v5.4 and SeqScape v2.7 softwares (Life Technologies).

### Shifted termination assays (STA)

STA is based on primer-extension methods where 3 oligonucleotides (2 for amplification and 1 for mutation detection) are used to detect a specific mutation. However, STA utilizes the incorporation of multiple labeled nucleotides to the detection primer as compared to incorporation of a single labeled nucleotide in other primer-extension-based methods. The detection primer anneals one base before the target site and is then extended only when target site is mutated. Extension of detection primer with multiple labeled nucleotides intensifies signal and increases PCR fragment length allowing mutation discrimination from wild type via peak color and fragment size after capillary electrophoresis [15].

We used Mutector II Mutation Detection Kits (TrimGen, Maryland) to identify mutations in codons 12, 13 and 61 of the KRAS gene which are the KRAS mutations reported to be most frequently involved in colorectal cancer [11]–[12], as well as specific mutations of the EGFR gene in characteristic locations in exons 18–21, including certain point mutations, deletions and insertions which identify patients who are most likely to respond to targeted lung cancer therapy, including tyrosine kinase inhibitors erlotinib and gefitinib. The Mutector II Mutation Detection Kits implement STA technology for the simultaneous detection and differentiation of mutations occurring on the same target site. Mutector II Mutation Detection Kit GP05-CM detects and differentiates all 12 mutations occurring in codons 12 and 13 while Mutector II Mutation Detection Kit GP06 is designed for 5 mutations occurring in codon 61. Mutector II Mutation Detection Kit GP07-02 detects mutations in characteristic locations in exons 18–21 of the EGFR gene. STA technology significantly improves sensitivity. It detects as low as 1% somatic mutations as compared to 15–20% via Sanger sequencing [15]. Briefly, 50 ng gDNA was PCR amplified using reagents provided via the PCR conditions described by the manufacturer. PCR products were cleaned up and mutations in codons 12, 13, and 61 were enriched separately by mutation specific primer extension reaction. Samples containing enriched mutations were cleaned from excess fluorescent dyes, diluted 5–10 times with water. Three microliters of the diluted primer extension products were mixed with loading buffer provided in the kit and fragment sized via capillary electrophoresis on ABI's 3500 Genetic Analyzer (Life Technologies). Capillary electrophoresis running conditions and instrument setup for data collection are explained in detail within the Mutector II Mutation Kit instructions manual.

### Functional prediction and molecular modeling of novel KRAS mutations

The functional impact of the non-synonymous (protein structure altering) exon 4 mutations was assessed using POLYPHEN-2 (polymorphism phenotyping v2) [16] and SIFT (sorting intolerant from tolerant tools) [17]. We also used the mutation assessor [18] to predict the functional impact of mutations based on evolutionary conservation of the affected amino acid in protein homologs. Additionally, molecular modeling was applied to predict the functional impact of the novel mutations detected. The K-ras protein structure used for modeling was obtained from the Protein Data Bank (PDB ID: 3gft). Modeling of mutations was performed and visualised using PyMOL [19]–[20]. PyMOL displays information on all steric interference between the mutated amino acid and other amino acid side chains and the configuration with the least steric interference is selected manually. The mutant side chains were modeled into positions of the protein using rotamers with lowest conflicting Van der Waals radii and the configuration with the least steric interference with other amino acid side chains. The wild type and the mutant protein structure were superimposed to highlight the predicted conformational changes caused by each mutation.

### Statistical analysis

Data analysis was performed using SPSS for windows version 17.0 (SPSS Inc., Chicago, IL). Results were cross-tabulated to examine the relationships between the variables. Statistical analysis for categorical variables was performed using χ-square for test of association and Fisher's exact test as appropriate. Where two continuous independent variables were examined, t-test and analysis of variance were used. A p-value of less than 0.05 is considered significant in all statistical analyses.

## Results

### Novel KRAS mutations

KRAS gene mutations were detected in the cancer tissue of 24 out of 56 cases studied (42.85%). Of these, 11 (19.64%) had exon 4 mutations localized between codons 134 and 150, while 13 (23.21%) had mutations in exon 2, affecting codons 12 and 13. Mutations were not detected in codon 61 of exon 3. The distribution of mutations in our cohort is shown in Table S2.

**Figure 1:**
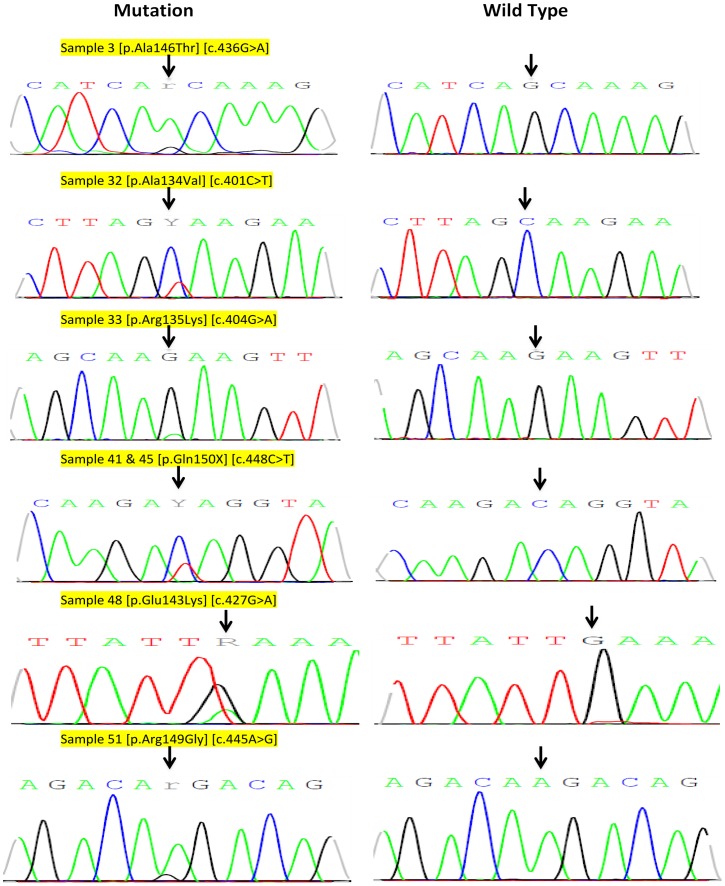
Images of Sanger sequencing electropherogram showing novel mutations (left panel) detected in the study cohort (reverse direction) and the corresponding wild type sequence (right panel) of normal tissue sections for the same patient indicating the somatic origin of detected exon 4 mutations. All illustrated mutations were confirmed via sequencing the forward strand. Sample number and mutation nomenclature according to HGVS guidelines are highlighted above each mutation. Arrows point to the location of base pair change.

**Figure 2:**
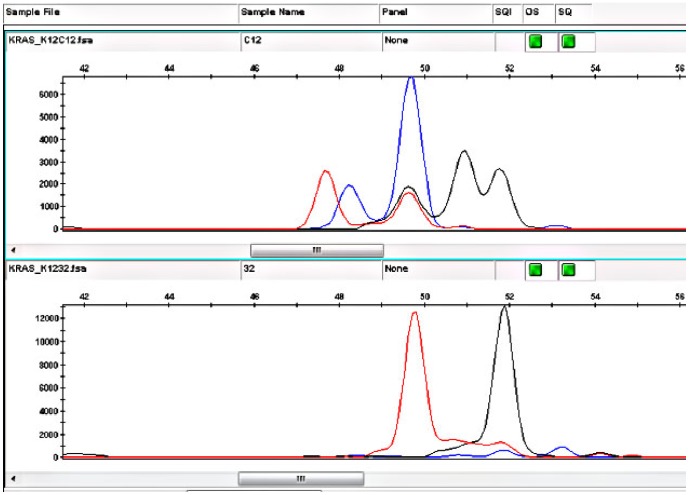
Shifted Termination Assay control for codon 12 mutations (top panel): 1st red peak: GGT>AGT; 1st blue peak: GGT>CGT; 2nd blue peak: GGT>GCT; 2nd red peak: GGT>GAT; 1st black peak: GGT>TGT; 2nd black peak: GGT>GTT; 3rd black peak: wild type. Bottom panel is for sample 32 showing the 2nd red peak (GGT>GAT) and the 3rd black peak (wild type).

The 11 cases with exon 4 aberrations harbored 8 different mutations (Table 2) of which one was previously reported (p.Ala146Thr, a missense mutation) and 7 were novel as revealed by the COSMIC database accessed on 19/09/2014. We confirmed all novel sequence variations detected in our cohort by sequencing the opposite strand. All samples showed an identical sequence variation in the opposite strand. The weak signal in sample 33 (Figure 1) could be attributed to lower tumor tissue content; the other (forward) strand also showed a weak signal of the sequence variation (c.404G>A) as well. However, sequencing the noncancerous tissue of the patient showed no signal whatsoever for variation c.404G>A indicating that the signal in the sample is above the detection limit of the sequencing protocol. One of the novel mutations (G138G) was detected in three patients and another (Q150X), detected in two other patients. Four of the seven mutations were missense mutations altering the amino acid sequence of the protein (A134V, R135K, E143K and R149G), whereas two mutation were synonymous (G138G and K147K) and one, a nonsense truncating mutation (Q150X). The missense and nonsense exon 4 mutations were observed in seven patients (12.5% of the total). Figure 1 is an electropherogram for the KRAS exon 4 nonsense and missense mutations that truncated or altered the KRAS protein amino acid sequence, respectively. Samples 32 and 45 also contained another mutation (p.Gly12Asp) in codon 12 of exon 2 as revealed by STA analysis, that was frequently detected in colorectal cancer patients (Figure 2). The novel exon 4 KRAS mutations were found to be somatic since sections of noncancerous colorectal tissue from patients who harbored those mutations showed the wild type sequence (Figure 1).

**Table 2:** Mutations detected in exon 4. Exon 4 of all samples was sequenced as described in text.

Sample Code	Codon Change	p.Mutant (1)	p.Mutant (3)	c.Mutant	Concomittant Exon 2 (Codon 12) Mutation	Comment
3	GCA>ACA	p.A146T	p.Ala146Thr	c.436G>A	none	previously reported
32	GCA>GTA	p.A134V	p.Ala134Val	c.401C>T	GGT>GAT (p.G12D, c.35G>A)	novel
33	AGA>AAA	p.R135K	p.Arg135Lys	c.404G>A	none	novel
41	CAG>TAG	p.Q150X	p.Gln150Stop	c.448C>T	none	novel
44	AAG>AAA	p.K147K	p.Lys147Lys	c.441G>A	none	novel
45	CAG>TAG	p.Q150X	p.Gln150Stop	c.448C>T	GGT>GAT (p.G12D, c.35G>A)	novel
48	GAA>AAA	p.E143K	p.Glu143Lys	c.427G>A	none	novel
50	GGA>GGG	p.G138G	p.Gly138Gly	c.414A>G	none	novel
51	AGA>GGA	p.R149G	p.Arg149Gly	c.444A>G	none	novel
53	GGA>GGG	p.G138G	p.Gly138Gly	c.414A>G	none	novel
61	GGA>GGG	p.G138G	p.Gly138Gly	c.414A>G	none	novel

Fifty six colorectal cancer tissue samples were analyzed. Only those with a mutation in exon 4 are illustrated in the table. All detected mutations were confirmed via sequencing the opposite strand. HGVS guidelines for mutation nomenclature were followed.

**Figure 3:**
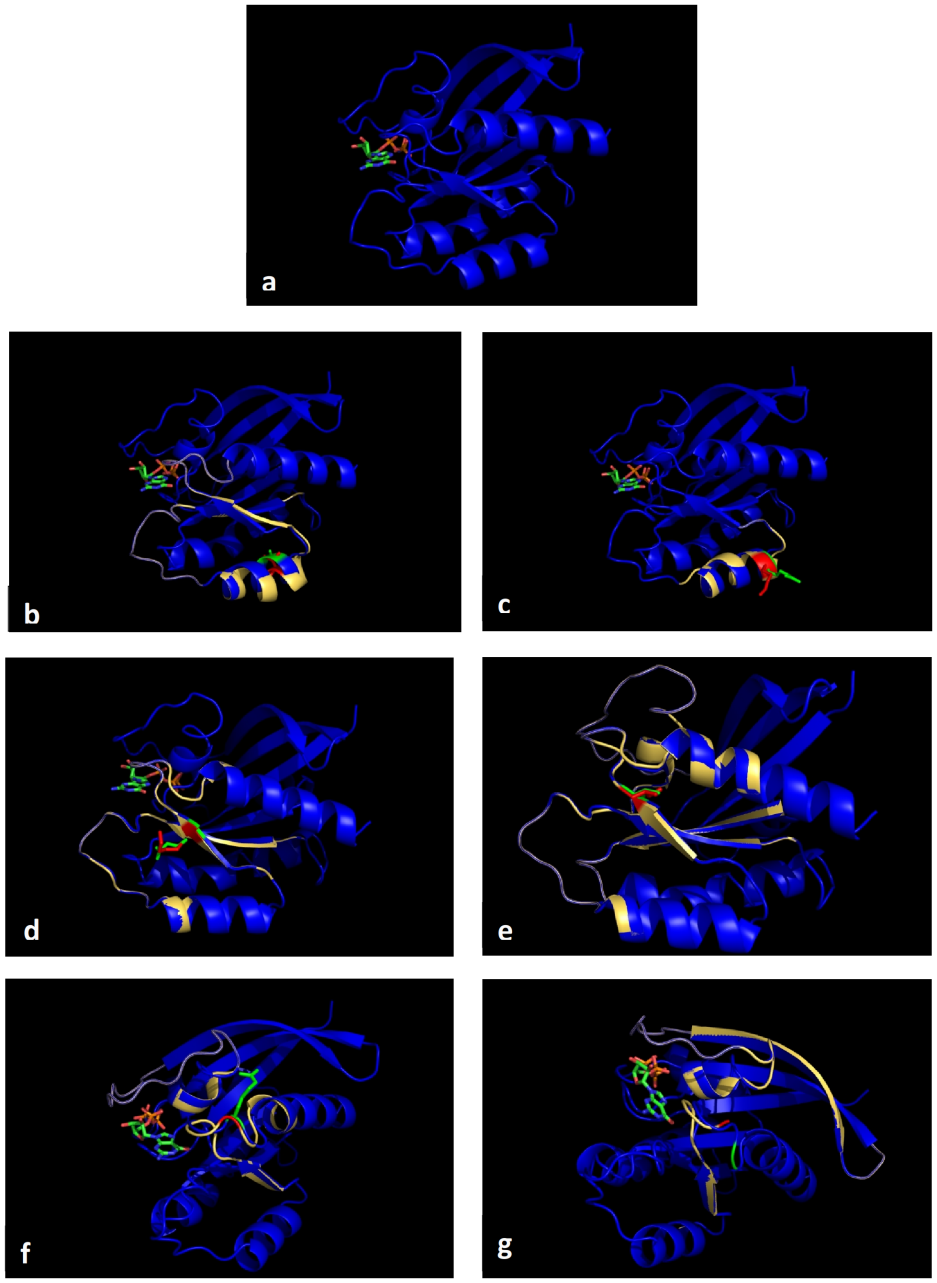
Predicting molecular modeling of KRAS mutations identified in patients with colorectal cancer. Wild type (WT) KRAS is shown in blue color and the mutant proteins are shown in yellow. The side chains of amino acids are shown in green for the WT residues and in red for the mutant residues. a) WT K-RAS showing side chain of A134 (green). b) Overlap of WT and mutant A134V KRAS showing predicted conformational changes caused by the A134V mutation (note the changes in the helix and beta sheet). c) Overlap of WT and mutant R135K KRAS showing predicted conformational changes caused by the R135K mutation (note the changes in the helix and loop chains) but doesn't affect the GTP binding pocket. d) Overlap of WT and mutant E143K KRAS showing predicted conformational changes caused by the E143K mutation (note the changes in the loop near the GTP binding pocket). e) Overlap of WT and mutant T144I KRAS showing predicted conformational changes caused by the T144I mutation (note the changes in the GTP binding pocket and the changes in the orientation of the bound GTP; green is wt and red is mutant). f) Overlap of WT and mutant R149G KRAS showing predicted conformational changes caused by the R149G mutation (note the changes in the helix and loop chains near the GTP binding pocket). g) Overlap of WT and mutant Q150X KRAS showing predicted conformational changes caused by the Q150X mutation (note the changes in the loop chain).

The functional impact of the non-synonymous mutations on the KRAS protein was assessed using bioinformatics tools and results are presented in Table 3. For example, the E143K and Q150X mutations are predicted to have a damaging and high impact on the protein whereas the R135K is predicted to be tolerated or to have neutral impact on the protein. Additionally we used molecular modeling to assess the functional impact of these novel KRAS mutations and results are shown in Figure 3. The molecular modeling data fit with the prediction from POLYPHEN-2 and SIFT, as well as conservation data (Table 3). For example, the R135K mutation is predicted to be benign and modeling also showed little predicted effect on protein structure. The remaining mutations are predicted to cause substantial changes in the protein structure in line with the predicted damaging effect by POLYPHEN-2.

**Table 3:** Molecular modeling data of KRAS exon 4 non-synonymous mutations.

Mutation	Polyphen-2 (score)	SIFT (score)	Mutation Assessor	protein domain affected
**A146T**	**Probably Damaging (1.000)**	**Deleterious (0.00)**	high	TURN
**A134V**	**Possibly Damaging (0.635)**	**Deleterious (0.00)**	medium	HELIX
**R135K**	**Benign (0.000)**	**Tolerated (0.73)**	neutral	HELIX
**E143K**	**Possibly Damaging (0.788)**	**Deleterious (0.01)**	high	STRAND
**R149G**	**Probably Damaging (0.989)**	**Deleterious (0.00)**	neutral	TURN
**Q150X**			N/A	HELIX

Point mutations specified by the **EGFR mutation** detection kit in exons 18, 20 and 21 of the EGFR gene were not detected in any of the 56 cases studied. InDel mutations in exons 19 and 20 were also not detected.

The main **clinical, pathological and immunohistochemical findings** in the reported cases are shown in Table 4. Tables 5 and 6 show a prevalence of female sex in cases with novel KRAS mutations compared to cases with other KRAS mutations and KRAS mutation negative cases (70%, 42.8% and 43.75%, respectively). They also show lower prevalence of lymph node metastasis and p53 expression although the differences are not statistically significant (p value ranging between 0.078 and 1.0). We did not find EGFR or HER2 protein expression, or EGFR gene mutations in any of the 10 cases. There was also no significant difference between the three groups regarding patient age, and tumor size, depth and grade.

**Table 4:** Clinical pathological and immunohistochemical findings in colorectal cancer cases with novel KRAS mutations.

Sample code	Case#	Age (years)	Sex	TumorGrade	Tumor size/cm	Tumor depth (pT)	Lymph node metastasis (N)	p53	EGFR	HER2
32	S05–2572	83	M	II	9	4	Present	−ve	−ve	−ve
33	S99–260	56	F	I	6	3	Absent	−ve	−ve	−ve
41	S99–2577	80	F	II	7	2	Absent	−ve	−ve	−ve
44	S98–3279	52	M	I	5.5	3	Absent	**+ve**	−ve	−ve
45	S02–3650	80	F	II	6	4	present	−ve	−ve	−ve
48	S02–780	37	M	II	7	3	Absent	−ve	−ve	−ve
50	S01–2003	58	F	II	2.5	3	Absent	−ve	−ve	−ve
51	S02–2717	48	F	II	4	3	present	**+ve**	−ve	−ve
53	S00–2675	61	F	II	3.5	2	Absent	−ve	−ve	−ve
61	S07–2242	39	F	II	5	3	Absent	−ve	−ve	−ve

**Table 5:** Clinical, pathological and immunohistochemical findings in colorectal cancer cases with novel K-ras mutations compared to cases with other K-ras mutations.

		All mutations N(%)	Novel mutations N(%)	p-value
Age (years)	Mean(SD)	59.1(14.7)	59.4(16.7)	0.656
Tumor size/cm	Mean(SD)	4.9(2.4)	5.5(1.59)	0.416
Tumor depth (pT)	1	1(7.1)	0(0)	0.733
	2	3(21.4)	2(20)	
	3	9(64.3)	6(60)	
	4	1(7.1)	2(20)	
Lymph node metastasis	Absent	9(64.2)	7(70)	0.591
	Present	5(35.7)	3(30)	
Sex	Male	8(57.1)	3(30)	0.129
	Female	6(42.8)	7(70)	
Tumor Grade	Grade I	1(7.1)	2(20)	0.706
	Grade II	12(85.7)	8(80)	
	Grade III	1(7.1)	0(0)	
P53	Neg.	9(64.2)	8(80)	0.683
	Pos.	5(35.7)	2(20)	
EGFR	Neg.	11(78.6)	10(100)	0.291
	Pos.	3(21.4)	0(0)	
HER2	Neg.	10(71.4)	10(100)	0.078
	Pos.	4(28.6)	0(0)	
Total Number		14 (100)	10(100)	

**Table 6:** Clinical, pathological and immunohistochemical findings in colorectal cancer cases with novel K-ras mutations compared to K-ras mutation negative cases.

		Mutation Negative N(%)	Novel mutations N(%)	p -value
Age (years)	Mean(SD)	57.2(14.9)	59.4(16.7)	0.491
Tumor size/cm	Mean(SD)	5.4(1.9)	5.5(1.9)	0.842
Tumor depth (pT)	1	0(0)	0(0)	
	2	5(15.62)	2(20)	
	3	22(68.75)	6(60)	
	4	5(15.62)	2(20)	0.877
Lymph node metastasis	Absent	17(53.12)	7(70)	
	Present	15(46.87)	3(30)	0.473
Sex	Male	18 (56.25)	3(30)	
	Female	14(43.75)	7(70)	0.277
Tumor Grade	Grade I	2 (6.25)	2(20)	
	Grade II	26 (81.25)	8(80)	0.251
	Grade III	4(12.5)	0(0)	
P53	Neg.	23 (71.87)	8(80)	
	Pos.	9 (28.12)	2(20)	1.000
EGFR	Neg.	23 (71.87)	10(100)	
	Pos.	9 (28.12)	0(0)	0.086
HER2	Neg.	25 (78.12)	10(100)	
	Pos.	7 (21.87)	0(0)	0.168
Total Number		32 (100)	10 (100)	

Three out of the five patients harboring the deleterious mutations had more advanced disease with increased tumor depth and lymph node metastasis (cases 32, 45 and 51), while two had localized disease (cases 41 and 48).

The two cases with concomitant exon 2 mutation (cases 32 and 45) showed greater tumor size and depth compared to most of the other cases with novel mutations and also had lymph node metastasis.

## Discussion

Activation of the KRAS oncogene has been implicated in colorectal carcinogenesis, being mutated in 30–40% of adenocarcinomas [5]–[8], a prevalence comparable to that observed in the present study (42.85%). The mRNA transcript of the KRAS gene is composed of 5765 bases coding for 188 amino acids. Exons 1, 2, 3, 4, and 5 contain 181, 122, 179, 160, and 5123 bases, respectively [21]. The majority of somatic mutations occur at codons 12 and 13 (situated in exon 2). Other less frequent mutations occur in exon 3 (codons 59/61) and exon 4 (codons 117/146) [22]–[23]. Approximately one third of colorectal cancers harbored mutations at the G12 and G13 codons while exon 4 mutations codon 117 and 146 were detected in only up to 5.5% of tumors [24]. Our study showed a much lower prevalence of exon 2 mutations (23.21%) and a much higher prevalence of exon 4 mutations (19.64%) with the exon 4 mutations localized between codons 134 and 150 rather than involving codons 117 and 146.

Exon 4 KRAS mutations are underestimated since all efforts of clinical testing focused on exon 2 (codons 12 and 13) and exon 3 (codon 61) mutations [22]–[25]. All seven novel KRAS mutations reported in the present article occurred in exon 4 and localized between codons 134 and 150. Thus we suggest including codons 134–150 as a hotspot for routine KRAS mutational analysis in colorectal cancer patients, rather than focusing only on codons 117 and 146. We detected missense and nonsense exon 4 mutations in 12.5% of the cases which is higher than what was previously reported, which amounted to 10% but also included exon 3 and NRAS in addition to exon 4 mutations [24]. All novel mutations were found to be somatic since all sections of noncancerous colorectal tissue from the 10 cases that harbored the mutations showed the wild type sequence.

The functional impact of the novel non-synonymous mutations on the KRAS protein was assessed using bioinformatics tools and molecular modeling (Tables 2 & 3, Figure 3). The R135K mutation resulted in the substitution of Arg for the similarly charged Lys that is predicted to cause little changes in interactions between adjacent residues. Bioinformatics tools showed that the R135K mutation is predicted to have a neutral effect on the protein. Similarly, molecular modeling showed a minor change to the protein structure caused by this mutation (Figure 3). In contrast, the E143K mutation is predicted to have a damaging effect on the protein by bioinformatics tools (Table 3) and by molecular modeling (Figure 3). This mutation resulted in the substitution of the negatively charged Glu for the positively charged Lys (Table 2) that is likely to cause major changes in the interactions of the adjacent amino acid residues. Figure 3 shows that the E143K mutation caused substantial changes to the structure of the KRAS protein especially in the loop near the GTP binding pocket. The R149G mutation is predicted to be neutral but molecular modeling showed that this mutation caused changes in the helix and loop chains near the GTP binding pocket. This is likely to be due to the changes in the interactions between amino acid residues caused by the substitution of the positively charged Arg for the non-polar Gly (Table 2). Additionally, this mutation is located near the conserved SAK motif and a recent study showed that a nearby mutation (K147E) results in a self-activating RAS protein that can act independently of upstream signals and exhibit a lower affinity for RAF kinase [26]. The Q150X mutation introduced a premature stop codon and is predicted to have an effect on the stability of the mRNA through nonsense-mediated decay resulting in reduced expression of the truncated protein. To the best of our knowledge, the Q150X mutation detected in the present study is the second KRAS truncating mutation to be reported in colorectal cancer in the literature. A KRAS mutation (CAG>TAG) determining a premature stop signal at codon 22 (Gln22Stop) has been previously found in a patient with metastatic colorectal cancer by Palmirotta, et al [27]. BRAF and p53 genes were not found to be modified and microsatellite instability was not present. The patient, however, was found to be unresponsive to an anti-EGFR treatment. Interestingly our two patients harboring the truncating mutation were EGFR negative by IHC. They also had no EGFR gene mutations. Several preclinical [28] and clinical [29] studies have shown that the occurrence of KRAS mutations in colorectal cancer is an independent predictive parameter of EGFR targeted therapy resistance. Finally, although the synonymous novel exon 4 mutations reported in the present study (including the G138G mutation that was detected in three patients) did not alter protein amino acid composition, they may still prove significant in tumorogenesis. There is growing evidence that synonymous mutations (often erroneously referred to as “silent”) can affect transcription, splicing, mRNA transport and translation, any of which could alter phenotype, rendering the synonymous mutation non-silent [30].

We detected one of the novel mutations in three cases (G138G) and another in two more cases (Q150X). Our sample size is rather small to conduct frequency analysis and comparison to larger published studies. However, the fact that these new mutations were seen in 5/56 cases indicates that further studies need to be done.

EGFR is over expressed in many types of cancers, especially colorectal cancer, and seems to reflect more aggressive histological and clinical behaviors [31]. It has also been shown that p53 protein over expression may help in potentially predicting metastatic spread to the lymph nodes in colorectal cancer [32]. Based on such information, the low prevalence of lymph node metastasis and p53 expression in our patients harboring the novel mutations coupled with the absence of EGFR and HER2 protein expression and EGFR gene mutations may generally suggest a low-grade pathway in colorectal cancer development in those patients, who are probably also resistant to anti-EGFR and anti-HER2 therapy. This speculation is in keeping with the finding that mutations in exon 4 of KRAS predict for a more favorable clinical outcome in patients with colorectal cancer [24]. Ironically, four of the seven novel exon 4 mutations detected in the present study are predicted to be deleterious to the KRAS protein as revealed by molecular modeling. Moreover, only three out of the five patients harboring the deleterious mutations had more advanced disease with increased tumor depth and lymph node metastasis (cases 32, 45 and 51), while two had localized disease (cases 41 and 48)! Could some of the mutations detected, alternatively, have a beneficial rather than a harmful effect on the host, possibly attributed to environmental factors? It has recently become clear that mutant RAS may result in highly divergent consequences in different tissues and environments [33]. For example, overexpression of HRasV12 in immortalized mouse NIH3T3 cells causes transformation associated with activation of Raf and PI3K pathways [34], whereas overexpression of HRas in normal fibroblasts causes a senescent-like cell cycle arrest [35]. Over expression of mutant RAS alleles results in a senescent-like phenotype that has been attributed to increased production of reactive oxygen species and associated stresses [36]–[37] and is likely unrelated to the normal functions of single copy mutant RAS, which results in tumor initiation without senescence [38]. It is to be noted that the two cases with concomitant exon 2 mutation (cases 32 and 45) showed greater tumor size and depth compared to most of the other cases and also had lymph node metastasis. The more extensive disease observed in those two cases may relate to the synergetic effect of the concomitant exon 2 mutation rather than a direct effect of the “deleterious” exon 4 mutations present. It is known that multiple mutations appear to be associated with a more aggressive disease [39].

The seemingly contradictory observations described above may, however, be due to the small sample size studied. It remains, therefore, that further more advanced KRAS testing, including next generation sequencing (NGS), on a large number of patients, particularly beyond the most common hotspot alleles in exons 2 and 3 is needed to explore the exact prognostic and predictive significance of the discovered novel mutations as well as their possible role in colorectal carcinogenesis.

Our discovery of novel Exon 4 KRAS mutations that are, so far, unique to Saudi patients from the Eastern Province may be attributed to environmental factors and/or genetic variation amongst different racial/ethnic groups. Alternatively, it may again be related to paucity of clinical studies on mutations other than those at codons 12, 13, 61 and 146 [12] and could, in the future, prove to be more frequent and non-race restricted.

The epidemiology of colorectal carcinoma in developing countries differs from that of developed countries. Colorectal carcinoma in developing countries, including those in the Middle East, is usually characterized by low incidence, young age of onset, left-sided location, poor differentiation, and paucity of precursor adenomas [40]–[48]. It has been suggested that environmental factors, especially lifestyle and dietary differences, play a major role in the observed epidemiologic differences. A study involving a number of Middle Eastern countries indicated that geographic variation in methylation also exists in colorectal carcinoma, possibly as a result of different environmental exposures [49]. Studies from various other countries have analyzed the frequency of the type of K-ras gene point mutation in colorectal cancer. Those studies were conducted in the UK [50]–[51], former Yugoslavia [52], Czech Republic [53], Norway [54], Switzerland [55], Mexico [56], USA [57] and The Netherlands [58]–[60]. All of the studies except that performed in former Yugoslavia [52] have identified the G>A transition as the most frequently found type of K-ras mutation. The pattern of specific alterations observed, i.e. G>A transitions and G>T transversions, could be due to differences in diet and/or other lifestyle factors. N-nitroso compounds, for example, in red and processed meat could induce G>A transitions [51] and this is supported by previous experimental studies [61]–[62]. A high intake of polyunsaturated fat, in particular linoleic acid, may be an important dietary risk factor for K-ras mutated colon tumors, possibly by generating G>A transitions or G>T or G>C transversions in the K-ras oncogene [11]. Interestingly, the meal that is mostly consumed in Saudi Arabia consists of lamb and rice.

In addition or as an alternative to environmental factors, the novel mutations detected may be attributed to genetic variation. Population-based studies have shown differences in colorectal cancer survival estimates that were reported to be higher in developed countries in comparison to less developed nations, with the exception of Eastern Europe [63]–[64]. Incidence rates in the United States have also shown clear racial/ethnic disparities for colorectal cancer. Incidence and mortality among Caucasians were lower than among African-Americans, but higher than among Asian and Pacific Islanders and Hispanics [65]. Five-year survival was found similar in non-Hispanic whites and Asian Americans [66]–[68]. It has been suggested that differences in the distribution of known/suspected risk factors account for only a modest proportion of the ethnic variation in colorectal cancer and that other factors, possibly including genetic susceptibility, are important contributors to the observed disparities [69]. It may be interesting to note that studies on breast cancer patients from the Eastern Province of Saudi Arabia revealed a spectrum of molecular breast cancer types that was in stark contrast with Western and other regionally based studies [70]. In a recent pilot study performed on Canadian and Saudi breast cancer patient populations, Amemiya, et al., using Next Generation SOLiD RNA sequencing and Ion Torrent exome targeted sequencing technologies, found a high prevalence for an SNV in FAM175A gene predicted to be deleterious in the Canadian as compared to the Saudi patients. In addition, a high prevalence of MSH6 gene deletions was seen in the Saudi patients, resulting in a frame shift in the Saudi population compared to the Canadian population [71].

## Conclusions

Our discovery of novel Exon 4 KRAS mutations that are, so far, unique to Saudi colorectal cancer patients from the Eastern Province may be attributed to environmental factors and/or racial/ethnic variations due to genetic differences. Alternatively, it may be related to paucity of clinical studies on mutations other than those in codons 12, 13, 61 and 146. Further, more advanced KRAS testing on a large number of patients of various ethnicities, particularly beyond the most common hotspot alleles in exons 2 and 3 is needed to assess the prevalence and explore the exact prognostic and predictive significance of the discovered novel mutations as well as their possible role in colorectal carcinogenesis.

## Acknowledgments

The authors acknowledge Dr Mohammad Al Hammad, Molecular genetics scientist, College of Medicine, University of Dammam for his critical revision and advice regarding molecular data. We also acknowledge Dr Eman El-Far, Biostatistician, College of Nursing, University of Dammam for performing statistical analysis.

## Supporting Information

Figure S1 **Immunohistochemical positivity in colorectal cancer.**
(TIF)

Table S1 **Sources and dilution of primary antibodies used in immunohistochemistry.**
(DOC)

Table S2 **Mutations in KRAS gene (exons 2, 3, & 4) and EGFR gene (delections in exon 19, insertions in exon 20, point mutations in exons 18, 20, & 21).**
(XLS)
