# Supplementary figures and images for: Novel KRAS Gene Mutations in Sporadic Colorectal Cancer
Source: PLoS One. 2014 Nov 20;9(11):e113350. doi: 10.1371/journal.pone.0113350 (PMC4239073; doi:10.1371/journal.pone.0113350)

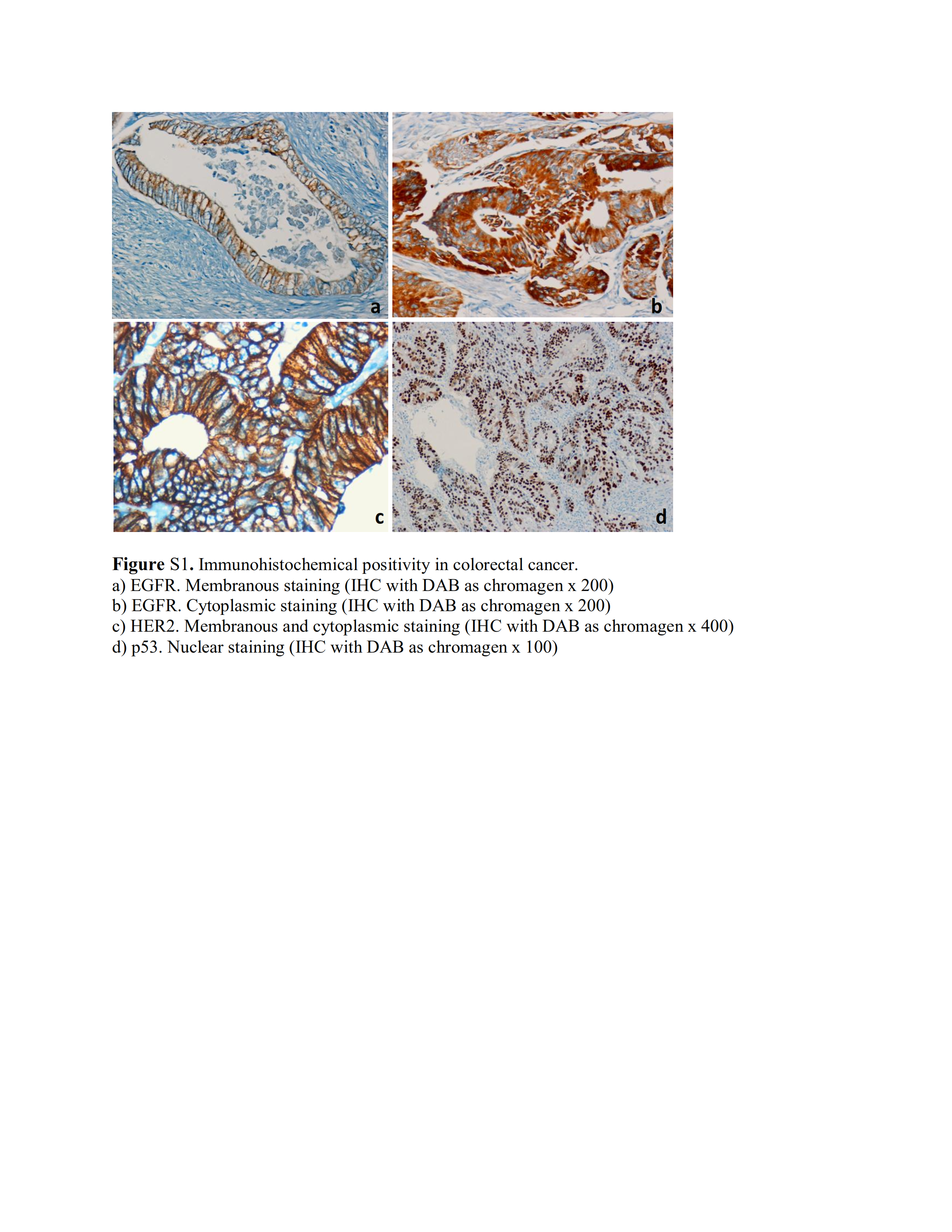

Supplement: Figure S1 — Immunohistochemical positivity in colorectal cancer. (TIF) [file pone.0113350.s001.tif]
