# Supplementary material for: Novel KRAS Gene Mutations in Sporadic Colorectal Cancer
Source: PLoS One. 2014 Nov 20;9(11):e113350. doi: 10.1371/journal.pone.0113350 (PMC4239073; doi:10.1371/journal.pone.0113350)
Supplement: Table S1 — Sources and dilution of primary antibodies used in immunohistochemistry. (DOC) [file pone.0113350.s002.doc]

**Table S1.** Sources and dilution of primary antibodies used in immunohistochemistry

| **Antibody** | **Clone** | **Manufacturer** | **Dilution** |
| --- | --- | --- | --- |
| HER2 | CB11 | Ventana | Prediluted |
| EGFR | H11 | Dako | 1:200 |
| P53 | D0-7 | Novacastra | Prediluted |
